# The cardiac nanoenvironment: form and function at the nanoscale

**DOI:** 10.1007/s12551-021-00834-5

**Published:** 2021-08-31

**Authors:** Jashan P. Singh, Jennifer L. Young

**Affiliations:** 1 Mechanobiology Institute, National University of Singapore, 117411 Singapore, Singapore; 2 Department of Biomedical Engineering, National University of Singapore, 117575 Singapore, Singapore

**Keywords:** Extracellular matrix, Topography, Ligand, Nanomaterial, Cardiomyocyte, Mechanosensitive

## Abstract

Mechanical forces in the cardiovascular system occur over a wide range of length scales. At the whole organ level, large scale forces drive the beating heart as a synergistic unit. On the microscale, individual cells and their surrounding extracellular matrix (ECM) exhibit dynamic reciprocity, with mechanical feedback moving bidirectionally. Finally, in the nanometer regime, molecular features of cells and the ECM show remarkable sensitivity to mechanical cues. While small, these nanoscale properties are in many cases directly responsible for the mechanosensitive signaling processes that elicit cellular outcomes. Given the inherent challenges in observing, quantifying, and reconstituting this nanoscale environment, it is not surprising that this landscape has been understudied compared to larger length scales. Here, we aim to shine light upon the cardiac nanoenvironment, which plays a crucial role in maintaining physiological homeostasis while also underlying pathological processes. Thus, we will highlight strategies aimed at (1) elucidating the nanoscale components of the cardiac matrix, and (2) designing new materials and biosystems capable of mimicking these features in vitro.

## Introduction

**Fig. 1:**
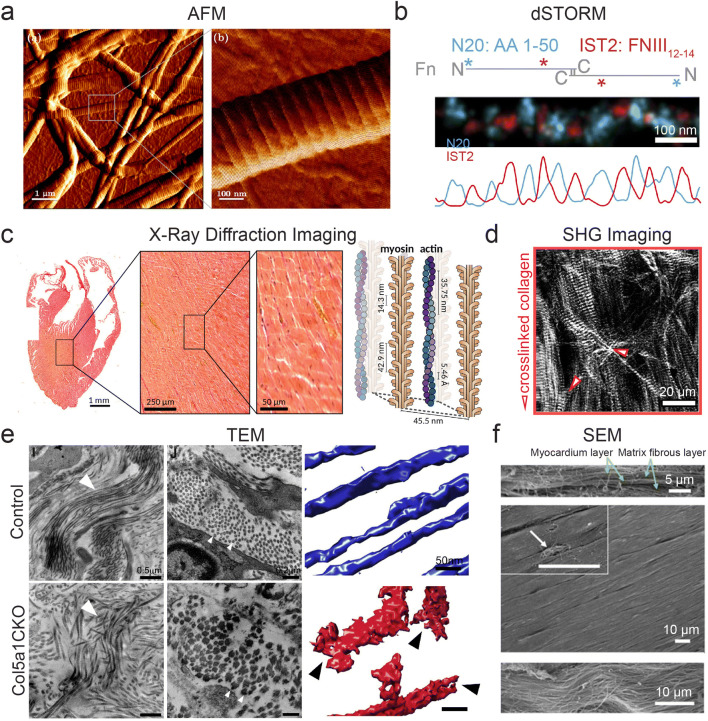
Methods for characterizing the cardiac tissue nanoenvironment. **a** AFM used to image a collagen type I fibril with diameter of ~300 nm with inset showing characteristic D-spacing (right panel). Adapted from Asgari et al. (2017) under CC BY 4.0. **b** Labelling periodic domains (N20:blue, IST2:red) of fibronectin protofibrils using direct stochastic optical reconstruction microscopy (dSTORM). Adapted from Früh et al. (2015) under CC BY 4.0. **c** Implementation of X-ray diffraction imaging for cardiac tissues and cells, showing resolution capability of heart sections down to the intracellular component scale (myosin, actin). Reproduced, with permission, from Nicolas et al. (2019). **d** SHG image of the myocardium with crosslinked collagen fibers causing disruption and rupture of myosin fibers (red arrows). Adapted from Nicolas et al. (2020) under CC BY 4.0. **e** Transmission electron microscopy (TEM) of scar area in animals with and without Col5a1 deletion in cardiac fibroblasts post-heart injury showing fibrillar disarray and alteration in fibril size in Col5a1CKO (left panel) and in cross section (middle panel) and corresponding electron tomogram (right panel). Reproduced, with permission, from Yokota et al. (2020). **f** SEM images of ex vivo adult rat myocardium at sideview (top panel), top view (middle panel), and magnified view (bottom panel) demonstrating aligned matrix fibers. Reproduced, with permission, from Kim et al. (2010)

The cardiac nanoenvironment, as we define in this review, comprises the nanoscale features of the extracellular matrix (ECM) with which cells interact. We will specifically focus on ECM organization and its nano-mechanical properties, identifying the functional consequences that alterations in the matrix have on cardiac cell function occurring in dynamic processes, e.g., disease and aging. The ECM not only provides structural support and signaling cues to its resident cells, but is also responsible for transmitting force between the cells of the heart in order to properly coordinate actuation (Hynes 2002; Parker and Ingber 2007). Many ECM properties are nanometer-scale in size, including topographical features, the distribution of cell-binding sites on protein fibers, and the diameter of the protein fibers themselves (Fig. 1a, b) (Asgari et al. 2017; Früh et al. 2015; Wallace et al. 2010). Furthermore, cells exert piconewton-magnitude forces on their surrounding matrix via integrin-based adhesions (Maynard et al. 2020), underscoring the importance of identifying the nanoscale features that play a role in maintaining such a sensitive force balance. To do this, we will first focus on the ECM and interactions cells make with it, with an emphasis on the tools that have been employed to uncover these nanoscale relationships. We will then discuss in vitro approaches aimed at mimicking the cardiac nanoenvironment before showing how these properties directly affect crucial cellular functions required to maintain cardiac homeostasis. While cardiac-specific studies are the main focus of this review, we also draw on findings from general matrix properties and nanoscale cell-matrix interactions that can be applied to the cardiovascular system.

## Nanoscale properties of the cardiac extracellular matrix

### Composition and organization

The cardiac ECM can be defined by two main regions: the basement membrane immediately surrounding cardiomyocytes (CMs), to which they directly interact via (mostly) integrins, and the interstitial matrix present between CMs, which provides structural support and houses the cardiac fibroblasts (Rienks et al. 2014). The basement membrane largely consists of fibronectin, type IV collagen, laminin, procollagens, hyaluronic acid (HA), and proteoglycans, while the interstitial matrix mainly consists of collagen types I and III (Chang et al. 2016; Rienks et al. 2014). Early studies on protein fibers using electron microscopy (EM) and X-ray scattering have uncovered the nanoscale structure and organization of these ECM units (James et al. 1991), while newer techniques harnessing super-resolution microscopy, e.g., single-molecule localization microscopy (SMLM), have focused on their molecular organization (Früh et al. 2015).

Collagen, being the most abundant protein of the heart, plays a predominant role in force transmission and in maintaining structural integrity (Bishop and Laurent 1995). Accordingly, alterations in its expression, abundance, and organization in diseased states and throughout aging result in severe consequences (Ozcebe et al. 2021; Yokota et al. 2020). Structurally, type I collagen forms large, rod-like fibers up to ~300 nm in diameter, while type III collagen forms smaller fibers closer to 100 nm in diameter (Fig. 1a) (observed by atomic force microscopy [AFM] in Asgari et al. 2017). These fibril-forming collagens are assembled into triple helical bundles with a characteristic overlap (termed D-period or D-spacing) occurring at periodic intervals of ~67–72 nm (as shown in Fig. 1a), which is believed to be the length scale at which cells interact with the molecule (Wallace et al. 2010). The exact distance between these periodic domains depends on tissue type, age, and disease state (James et al. 1991; Wallace et al. 2010). While collagen types I and III are the most abundant in the heart, there are other collagen types that also have important functions in both ECM homeostasis and repair. For instance, collagen type VI has been shown to be upregulated in aging hearts (Ozcebe et al. 2021). This collagen is highly flexible, exhibiting an arc curvature of up to 120° and with a characteristic periodicity of globular domains of ~85 nm (Lansky et al. 2019).

Fibronectin, whose fibrils are ~2 nm wide, plays an important role in both heart morphogenesis and in the fibrotic response post-injury (Erickson et al. 1981; Konstandin et al. 2013; Mittal et al. 2013). The nanoscale structure of fibronectin has been studied extensively, with a particular interest in how the molecule unfolds under force, thereby exposing cryptic binding domains (Erickson 2002; Szymanski et al. 2017). Depending on the tensional state of fibronectin, changes in ligand binding site accessibility, growth factor/cytokine attachment, and ECM organization can occur, all directly affecting cellular behavior (Vogel 2018). As the tensional state of the fibronectin molecule is correlated with tissue transformation (either healthy transformation that occurs in development or in loss-of-function processes such as aging), investigation into fibronectin organization at the nanoscale is important. In one approach, the molecular organization of individual fibrils was elucidated using site-specific labeling combined with SMLM, in which different N- and C-terminal epitopes exhibit periodicities ranging from 60 to 130 nm, or ~95 nm on average (Fig. 1b) (Früh et al. 2015). This closely matches early studies using EM and immunogold labeling of the type III homology sequence EIIIA site, in which a periodicity of ~84 nm was identified (Dzamba and Peters 1991).

Uncovering the nanoscale architecture of proteins has led to the understanding of how cells interact and arrange specific ECM components. Using cryo-scanning transmission electron tomography (CSTET) for collagen type VI and fibronectin in fibroblast cultures has shown that these proteins organize in distinct conformations and interact with surrounding cells in fundamentally different ways. Particularly, fibronectin was observed covering fibroblasts in a branching and merging pattern parallel to the cell axis while collagen type VI was found in open lattice-like networks consisting of interconnected polygons on the order of a few hundred nanometers in size arranged further from the cell body (Lansky et al. 2019). The nanoscale properties of other ECM components beyond collagen and fibronectin are equally important for cardiac function. In various physiological and aberrant processes of the heart, ECM composition and organization have been shown to change with development, disease, and age (Jallerat and Feinberg 2020; Ozcebe et al. 2021) (and recently reviewed by Frangogiannis 2019; Rienks et al. 2014; Silva et al. 2021). For instance, young cardiac ECM is predominantly composed of type I collagen, followed by laminin and type IV collagen, while aged ECM is predominantly composed of type VI collagen, followed by collagen types I and IV (Ozcebe et al. 2021). It has also been shown that laminin is one of the main integrin binding partners in the adult heart, with nanoscale domains ranging from ~15 to 50 nm depending on isoform (Yurchenco et al. 1997). To further complicate the issue, heterotypic assembles of proteins can alter not only fibril size, but also the nanoscale distribution of ligands to which cells attach, e.g., fibrils containing both type I collagen and fibronectin, exhibit a periodicity of 67 nm vs. the 84 nm observed in fibronectin fibrils alone (Dzamba and Peters 1991).

While investigations into cell-ECM interactions at the nanoscale in whole tissues is still lacking, methods for more comprehensive ECM characterization at sub-micron length scales have advanced in recent years. Scanning X-ray diffraction optics have improved, leading to the development of nano-focused hard X-rays for studying cardiac tissue, albeit with some structural degradation issues arising from the single beam exposure (Fig. 1c) (Nicolas et al. 2019, 2020). Second harmonic generation (SHG) imaging has also allowed for the visualization of individual collagen fibrils at sub-micron scale in heart tissue, revealing a tight correlation between CM myofibril disruption and altered collagen crosslinking in hypertrophic hearts, an observation that had not previously been reported due to resolution limitations (Fig. 1d) (Nicolas et al. 2020). To our knowledge, this was the first study to demonstrate that altered nanoscale properties of the ECM directly play crucial roles in cellular function within tissues, an important first step in understanding the effects of the cardiac tissue nanoenvironment on heart function. Transmission electron microscopy (TEM) has uncovered nanoscale alterations in matrix organization as a function of collagen production, with a collagen type V knockout model exhibiting fibrillar disarray and increased fibril diameter post-myocardial infarction (MI) (Fig. 1e) (Yokota et al. 2020). Other approaches have harnessed laser microdissection (LMD) followed by mass spectrometry (MS) to analyze components of specific cardiac nanoenvironments, e.g., amyloid plaques in transthyretin amyloidosis (ATTR), although losing precise spatial information (Kourelis et al. 2020).

Matrix nanotopography is another important aspect to consider, as topography has been shown to dictate cellular morphology, interactions with other cells, and cell migration via contact guidance (Afzal et al. 2014; D.-H. Kim et al. 2012). Using scanning electron microscopy (SEM) on ex vivo myocardial tissue, ~100-nm diameter fibers were found to organize in parallel, providing directional support for the characteristic anisotropic organization of muscle fibers (Fig. 1f) (Kim et al. 2010). Many studies have focused on determining collagen orientation in injured hearts, from early methods using polarized light microscopy (Whittaker et al. 1989) to more recent approaches using computational models for describing the spatial heterogeneity observed during infarct remodeling (Richardson and Holmes 2016). As collagen secretion and crosslinking increase, not only are binding sites altered, but matrix topography itself undergoes changes. At the individual integrin level, nanotopography has been shown to influence mechanosensitive signaling processes in cells (Chighizola et al. 2020). These findings demonstrate an integrin clustering dependence on nanotopography in nascent adhesion formation, ultimately driving the force loading response in cells—a clear cut example of small features driving changes at a length scale an order of magnitude higher.

### Mechanical properties

There are two primary mechanical aspects of the nanoenvironment: stiffness and the tensional state of protein fibers. While several transmembrane molecules mediate the attachment of cells to their surroundings, the most ubiquitous and well-studied are integrin-based adhesions (Howard and Baudino 2014). Integrins, whose individual unit size is on the order of tens of nanometers, interact with specific binding domains in proteins and transmit mechanical information to cells and the matrix in a bidirectional manner (Hynes 2002; Ross 2004). Due to the small-scale nature of these interactions, even tiny alterations in ECM mechanics can lead to large effects on cellular function. At the nanoscale, tissue elasticity has been measured using AFM, in which nanometer-sized probes are used to apply piconewton levels of force, which are on the order of magnitude that cells experience. The elasticity of the cardiac ECM has subsequently been shown to change throughout development, myocardial injury, and disease (Berry et al. 2006; Young and Engler 2011).

During cardiac remodeling post-injury, drastic changes occur in the ECM, largely via activated cardiac fibroblasts that secrete the majority of the matrix, in order to mitigate structural damage following cardiac cell death (Dobaczewski et al. 2010). Protein composition is altered via an increased production of collagen, particularly type I, which is stiffer than type III, along with other components like fibronectin (Asgari et al. 2017; Konstandin et al. 2013). Furthermore, lysyl oxidases are upregulated, which initiate crosslinking of collagen fibrils (Al-u’datt et al. 2019; Mäki 2009). The combination of both leads to a fibrotic scar that has reduced contractility, resulting in heart stiffening, altered electrical signal propagation, and reduced cardiac performance (Al-u’datt et al. 2019; Miragoli et al. 2007; Murtha et al. 2017; Richardson et al. 2015). Indeed, such changes in mechanical properties have been shown to affect myofibril maturation and contractility of CMs, with impairment of both observed on matrices of pathophysiological values (>50 kPa representing diseased vs. ~10 kPa representing healthy) (Engler et al. 2008; Jacot et al. 2008; McCain et al. 2014).

Nanoscale mechanics at the single protein level have been extensively studied on fibronectin to understand how small forces affect ligand accessibility and concomitant cell function (Arnoldini et al. 2017; Cao et al. 2012; Diao et al. 2010; Ohashi et al. 1999). As fibronectin is well-known to harbor force-dependent cryptic binding sites (Erickson 2002), the tensional state of the protein has been of particular interest, and has been studied using a variety of techniques, including phage display for tensile state-specific peptide binding (Cao et al. 2012), bacterial binding domain nanoprobes specific to the relaxed state (Arnoldini et al. 2017), and molecular dynamics simulations of binding accessibility vs. conformation (Diao et al. 2010). Interestingly, fibronectin fibers have been shown to exist in a tensed state in healthy tissues, whereas diseased tissues exhibit a more relaxed, unstretched state (Arnoldini et al. 2017). Using the tension-sensitive bacterial adhesin-derived peptide FnBPA5, which binds specifically to relaxed fibronectin, fibers were found to be distributed throughout the healthy heart in a tensed configuration as compared to that of the tumor stroma, which was more heterogeneous and exhibited an abundance of relaxed fibronectin (Fonta et al. 2020). As the tumor stroma shares similarities with fibrotic tissue, the tensional state of fibronectin is likely to be comparably affected in myocardial injury and disease.

### Nanoscale interactions between cells and matrix

CMs attach to the basement membrane through assemblies called costameres, where their binding is mediated by integrins, integrin-related proteins, and dystrophin-glycoprotein complexes (DGC) (Sit et al. 2019). Through these sites, CMs sense matrix rigidity via parallel machinery regulated by both muscle and non-muscle myosin contractions (Pandey et al. 2018). In turn, the force transmitted between CMs and the surrounding ECM affects cellular contractility and myofibrillogenesis (Chopra et al. 2018; Danowski et al. 1992; Sit et al. 2019). The same principles apply for cardiac fibroblasts, where integrin-based focal adhesion complexes mediate attachment and force transmission from the interstitial matrix, driving their phenotype and activation state (Manso et al. 2006).

Integrin expression has been shown to vary in both heart development and disease, as well as among different cell types (Israeli-Rosenberg et al. 2014; Ross 2004). In CMs, the integrin subtypes most highly expressed are α1β1, α5β1, and α7β1, which are mainly binding receptors for collagen, fibronectin, and laminin, respectively (Israeli-Rosenberg et al. 2014). However, predominant subtypes have been shown to change based on developmental stage and disease state (Brancaccio et al. 2009; Nawata et al. 1999). This is important to note as different integrin heterodimers have been shown to play differential, yet complementary roles in rigidity sensing. For example, on fibronectin-based microenvironments, α5β1 integrins are responsible for force generation, while αv-class integrins facilitate structural adaptation to force (Schiller et al. 2013). Furthermore, the nanoscale spacing of integrins has been shown to dictate the amount of tension they can sustain, with integrins spaced 100 nm apart displaying significantly reduced tension vs. those spaced 50 nm apart (Liu et al. 2014). As previously mentioned, ligand-binding sites can be altered in various biological processes; therefore, it is logical to conclude small alterations in the nanoenvironment can cause drastic alterations in cellular behavior. Indeed, a slew of mechanosensitive pathways have been shown to play an important role in myocardial function, many of which depend on small-scale ECM properties (Ward and Iskratsch 2019).

While the visualization of nanoscale integrin-based attachments to the ECM remains a challenge, advancements in cryo-electron tomography have allowed for the ultrastructural identification of focal adhesion complexes. On fibronectin, focal adhesions in fibroblasts were shown to assemble into particles of ~25 nm in size with an average interspacing of ~45 nm. Furthermore, it was demonstrated that this architecture is highly dependent on actin contractility, as treatment with a Rho-kinase inhibitor, Y-27632, caused rapid reduction in focal adhesion size by ~60% (Patla et al. 2010). 3D super-resolution microscopy has also been harnessed to determine the molecular architecture of focal adhesion complexes (Kanchanawong et al. 2010). The interconnectedness of the various properties pertaining to the tissue nanoenvironment highlighted here (composition, mechanical properties, and cell-ECM interactions) was emphasized in a recent study on the role of type V collagen in cardiac tissue remodeling. When type V collagen, which is minimally expressed in the heart post-injury, was knocked out, the scar that forms post-injury is ~15% softer than a wildtype scar. This results in enhanced expression of αvβ3 and αvβ5 in the activated myofibrolasts, causing a deregulation of ECM production, eventually leading to scar expansion and increasingly poor cardiac function (Yokota et al. 2020). Mitigation of the severe scar phenotype can be achieved by treatment with Cilengitide, a specific inhibitor of αvβ3 and αvβ5 integrins, highlighting the feasibility of tissue nanoenvironment modification as a treatment strategy for MI (Yokota et al. 2020).

## In vitro systems recapitulating the cardiac nanoenvironment

Due to the difficulties in measuring and quantifying the influence of nanoscale matrix features on cells in vivo, engineered materials have been designed to allow for the study of cellular responses to nanoscale properties in vitro. Since the first demonstration that cells interact with nanotopographical features of their surroundings back in 1991 (Clark et al. 1991), the field of nanoengineered materials has made huge advancements in the development of bio-inspired nanoscale substrates and the subsequent study of cellular responses to them (Cavalcanti-Adam et al. 2006; Dalby et al. 2007; Schvartzman et al. 2011). This section will focus on the various material systems that have been developed specifically to study the tissue nanoenvironment and how cells are influenced by such properties.

### Nanotopography

**Fig. 2:**
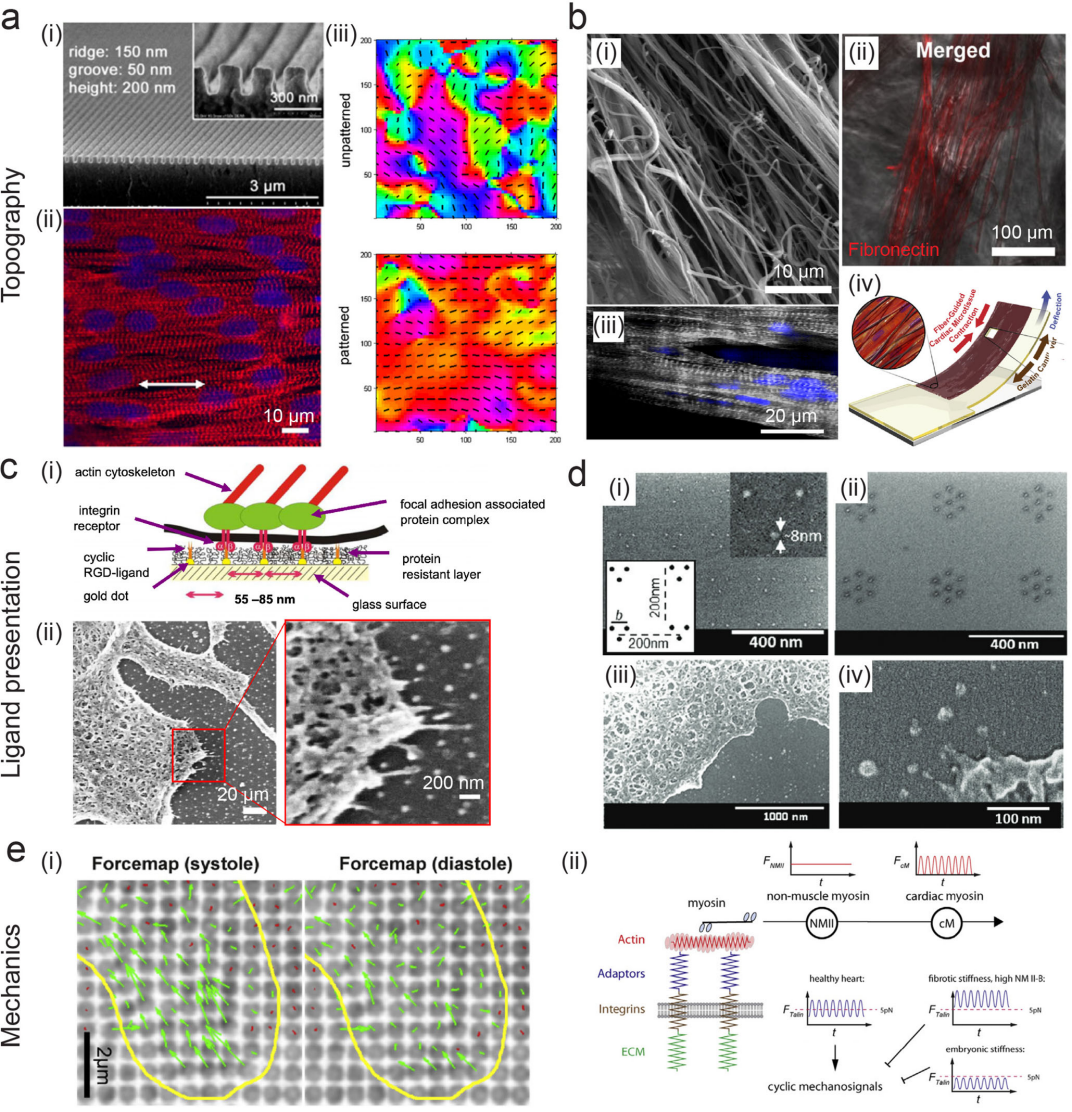
In vitro platforms for interrogating nanoscale cardiac properties. Three properties of the cardiac nanoenvironment are highlighted: topography (**a**, **b**), ligand presentation (**c**, **d**), and mechanics (**e**). **a** (i) Nanotopography mimicking cardiac matrix fibers show (ii) enhanced alignment and striations of neonatal rat ventricular myocytes (NRVMs) (actin:red, nuclei:blue) and (iii) improved directional contractility on patterned (bottom) vs. unpatterned (top) substrates, indicated by the color heatmap and vector field. Reproduced, with permission, from Kim et al. (2010) **b** (i) SEM image of synthetic nanofibers. (ii) Fibronectin (red) deposits on the fibers and (iii) CMs align along fibers (α-actinin:grey, nuclei:blue). (iv) Contractile stress is measured using a fiber-coated gelatin microphysiological system via cantilever defection. Reproduced, with permission from Ahn et al. (2018). **c** (i) Schematic of BCML for controlling ligand presentation with nanopatterns and (ii) SEM image of a cell interacting with an 80-nm pattern. Reproduced, with permission, from Hirschfeld-Warneken et al. (2008). **d** Controlling ligand presentation using a modified NIL technique for producing nanodot arrays of various geometries—shown here clusters of (i) three and (ii) seven, with cell interactions in (iii) lower and (iv) higher magnification. All are SEM images. Reprinted, with permission, from Schvartzman et al. (2011). **e** (i) Force maps of NRCMs (outlined in yellow) on nanopillars during systole (left) and diastole (right) with displacements indicated in green. (ii) Proposed model of CM matrix rigidity sensing using non-muscle and muscle myosin contractions, which work in series. Adapted from Pandey et al. (2018) under CC BY 4.0

To understand the increasingly complex in vivo cardiac tissue nanoenvironment, researchers at the forefront of nanotechnology have designed various materials to study the impact of topography and spatial organization of ligands, both in static and dynamic conditions. In order to mimic nanotopographical matrix features, approaches have been undertaken using both nanolithographic techniques and nanofiber fabrication methods. A seminal study demonstrating the importance of nanotopographic cues in regulating cardiac function showed that by employing rational design aimed at mimicking the nanofibrous myocardial matrix observed in SEM, cardiac contractility and coupling could be enhanced (Fig. 2a) (Kim et al. 2010). To do this, the authors used UV-assisted capillary lithography-based nanomolding to create poly(ethylene glycol) substrates with nanoridges and grooves ranging from ~50 to 800 nm. They found cell-cell coupling and conduction velocity to be a function of cell penetration into the nanogrooves, dependent on nanoscale parameters (Kim et al. 2010). A similar platform was later used to examine structural maturation of human-induced pluripotent stem cell-derived CMs (hiPSC-CMs) with a nanogrid culture array comprised of multiple nanogrooved topographies functionalized with Arg-Gly-Asp (RGD) peptides, finding an optimal CM sarcomere length at ~800-nm nanogroove width (Carson et al. 2016). Biomimetic nanotopographical surfaces have also been employed in the study of cardiac diseases such as MI, in which the spatial organization of ECM fibers has been shown to be altered post-injury (Goergen et al. 2016). By generating materials with random and aligned collagen nanotopography, a link between spatial variation of collagen fibers post-MI was shown to affect activated myofibroblast heterogeneity (Bugg et al. 2020).

In another strategy, electrospun nanofiber composites have been developed to mimic the fibrous nature of cardiac ECM (reviewed elsewhere: Capulli et al. 2016; Kim and Cho 2016). Fiber size, mechanical properties, and orientation can all be tuned to match specific nanoscale matrix properties (Kai et al. 2011; Kumar et al. 2020). As the cardiac ECM is comprised of parallel fiber bundles, anisotropy is an important matrix feature to recapitulate. Using a poly(ε-caprolactone) (PCL)/gelatin composite, aligned nanofibrous electrospun scaffolds with fiber diameters of ~250 nm were produced, exhibiting anisotropic wetting and mechanical characteristics matching that of native tissue (Kai et al. 2011). When cultured on these substrates, rabbit CMs showed enhanced alignment and spreading on parallel vs. random fibers, highlighting the importance of nanoscale contact guidance (Kai et al. 2011). Mechanical pulling has also been employed to create anisotropic nanofibers, which support the formation of aligned, contractile cardiac microtissues (Fig. 2b) (Ahn et al. 2018). Furthermore, mimicking the native ECM in 3D using PCL-aligned nanofiber scaffolds demonstrated the critical role nanoscale matrix features play in development, as differentiation of stem cells into functional CMs was enhanced by these scaffolds (Ding et al. 2020).

Cells exist in a dynamic environment, which is especially true for contractile CMs, where topographical matrix features do not remain in a static state. Thus, numerous studies have aimed to mimic the spatiotemporal reorganization of ECM features in vitro (Kumar et al. 2019; Sun et al. 2020; Young and Engler 2011). Using a PCL shape memory polymer (SMP), nanogrooves were designed that transition their orientation by 90° upon heating. The effect of temporal changes in anisotropic nanotopography was observed on primary CMs, in which alterations in cell alignment, contraction orientation, and focal adhesions occurred post shape transition (Mengsteab et al. 2016). A similar approach was used to observe time-dependent myofibril reorganization of hiPSC-CMs via SMP-coated polyelectrolyte multilayers yielding flat to wrinkled topographies, underscoring the importance of precisely coordinated cues in cardiac development (Sun et al. 2020). Furthermore, dynamic changes in nanotopography were shown to regulate two well-known Hippo pathway effector proteins, yes-associated protein (YAP) and transcriptional co-activator with PDZ-binding motif (TAZ), which have previously been identified in adult cardiac progenitor cell mechanosensing and fate decision (Gise et al. 2012; Xin et al. 2011). Using this system, YAP/TAZ were shown to shuttle between the nucleus and cytoplasm in response to dynamic modifications of the substrate nanostructure, highlighting the fine mechanosensitivity of CMs (Mosqueira et al. 2014).

### Ligand presentation

Controlling ligand presentation at the nanoscale requires the implementation of precise lithographic techniques due to the small-scale nature of such interactions. A variety of methods have been described to achieve defined nanoscale cell-ligand interactions, mostly employing self-assembly or nanolithography (Glass et al. 2003; Schvartzman et al. 2011). Block copolymer micelle nanolithography (BCML) has been utilized for arranging metallic nanoparticles onto substrates, to which cell-adhesive peptides can be directly linked, with nanometer control over interparticle/ligand spacing (Fig. 2c) (Glass et al. 2003; Hirschfeld-Warneken et al. 2008). Numerous studies have shown that cell adhesion (Arnold et al. 2004), migration (Cavalcanti-Adam et al. 2006), force loading (Oria et al. 2017), and drug sensitivity (Young et al. 2020) are dependent on small (~tens of nanometers) changes in ligand spacing. Furthermore, this technique has identified how cells sense both physical and spatial nanoscale matrix features by recruiting more integrins to distribute force (Oria et al. 2017). Top-down lithographic techniques, such as e-beam lithography (EBL) and nanoimprint lithography (NIL), have also been employed to create metal-based patterns with customizable features. Studies employing EBL and NIL have identified the existence of a minimal matrix adhesion unit size for fibronectin that supports cell adhesion and spreading (Fig. 2d) (Schvartzman et al. 2011), as well as have shown that the spatial arrangement of integrin nanoclusters depends on matrix fiber organization (Changede et al. 2019). This has important implications in cardiac matrix remodeling that can occur with age or injury post-MI, as fibrosis results in the production of large fibers, thereby reducing the ability of the cell to remodel the matrix (Changede et al. 2019).

In another approach, DNA origami nanostructures have been utilized for controlling nanoscale ligand organization, as they can be designed to display a specific ligand number, spacing between ligands, and multivalency (Hawkes et al. 2019). To this end, structures with 6 or 12 RGD peptide ligands, equivalent to interligand spacings of 60 or 30 nm, respectively, were immobilized 200 or 300 nm apart to observe the effects of local vs. global ligand concentration on neonatal rat CMs (NRCMs). While overall cell area and F-actin content increased with increasing local and global concentration of ligands, significant differences in adhesion of NRCMs could be observed when altering the global ligand concentration while maintaining local concentration. This indicates that the density between ligands regulates CM adhesion, yet exactly which integrin subunits are responsible for this response remains to be elucidated (Hawkes et al. 2019).

### Nanomechanics

Mechanical properties of the matrix influence cardiac cell behavior in development, homeostasis, and disease. This necessitates in vitro tools for both controlling and measuring forces cells experience at the matrix in order to quantify CM mechanotransduction (reviewed by Chin et al. 2019; Yadav et al. 2021). To study how CMs measure rigidity at single adhesion sites, a nanopillar platform was employed to correlate nanometer displacements of cells to their exerted forces (Fig. 2e) (Pandey et al. 2018). This setup identified that CMs sense simultaneous cardiac and non-muscle myosin contractions, with talin, a mechanosensitive unit within the cell, being stretched in an oscillating manner on physiological substrate rigidity vs. no tension on embryonic stiffness and continuous tension on a fibrotic stiffness. As tissue mechanics, CM contractile machinery, and adhesion sites are all altered in cardiac disease/injury, altered CM function can occur via aberrant mechanotransduction (Fig. 2e) (Pandey et al. 2018).

Approaches for measuring piconewton-scale forces exerted by cell surface receptors have harnessed tension probes (e.g., molecular tension-based and DNA-based), optical tweezers, as well as AFM (Krieg et al. 2019; Li et al. 2017; Liu et al. 2014; Rao et al. 2020). Due to their sensitivity, tension probes can quantify the amount and localization of integrin tension during cell attachment and spreading, while AFM-based techniques can measure single molecule mechanics as a function of applied force. Such techniques have been coupled with nanoscale matrix features, e.g., ligand spacing and nanotopography, demonstrating that adhesion formation and force sensing exhibit nanoscale sensitivity to ligand presentation (Chighizola et al. 2020; Liu et al. 2014). Small variations in nanoscale matrix features have also been shown to directly affect cellular-level tension as measured by traction force microscopy on soft nanopatterned hydrogels (Oria et al. 2017). Force loading can be explained by a modified molecular clutch model, in which clutches (i.e., integrins) reach a threshold force where they can no longer be recruited to stabilize adhesions, thereby resulting in adhesion collapse. This occurs at a critical ligand spacing and substrate rigidity, which can explain how altered mechanotransduction in diseased cardiac tissue leads to aberrant cardiac cell function, as both tissue mechanics and composition become transformed (Oria et al. 2017; Yokota et al. 2020).

## Conclusions and perspectives

**Fig. 3:**
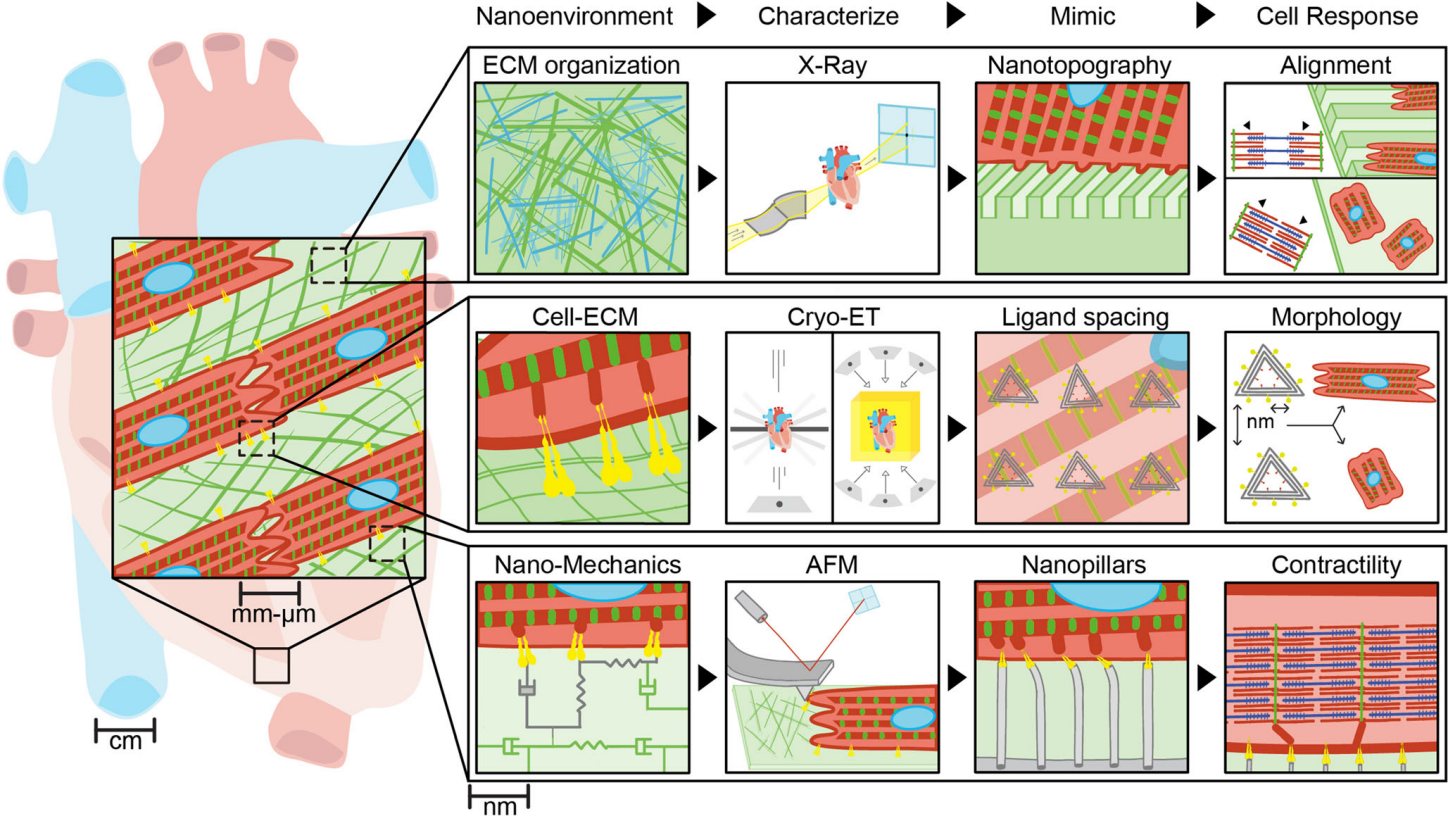
Overview of the cardiac tissue nanoenvironment. Here we summarize components of the tissue nanoenvironment and identify examples of methods discussed in this review to characterize these properties at the nanoscale, in vitro tools developed to recapitulate ECM features, and cellular responses that rely on nanoscale matrix cues

Cardiovascular mechanobiology has uncovered new insights into how subcellular forces, matrix cues, and signaling molecules guide cardiac development, homeostasis, and disease progression. While contributions exist across multiple length scales, here we focused particularly on nanoscale features of the extracellular environment. We have defined such features (matrix composition, organization, mechanics, and ligand interactions) and summarized the high-resolution imaging techniques that have identified these small-scale properties. We then outlined in vitro systems for controlling nanoscale tissue properties using new materials design and described the resulting cellular responses (summarized in Fig. 3). While we mainly focused on aspects of the ECM, it is important to note that other nanoscale features of the cardiac system are equally important. Many studies have identified subcellular structures in muscle cells that exhibit nanometer-sized features and are critical to their proper function, including the sarcoplasmic reticulum (Rog-Zielinska et al. 2021), the cardiac couplon (Jayasinghe et al. 2018), and sarcomeres (Wang et al. 2021). Furthermore, there are numerous cutting-edge techniques not mentioned in this review that could unravel the complexities of the cardiac nanoenvironment, including, but not limited to, serial block face SEM (Pinali et al. 2013), DNA-paint (Schnitzbauer et al. 2017), localization AFM (Heath et al. 2021), AFM-based simultaneous topography with recognition imaging (TREC) (Chtcheglova and Hinterdorfer 2018), as well as various super-resolution microscopy techniques (e.g., VividSTORM) (Barna et al. 2016). We are only at the tip of the iceberg of understanding how nanoscale features affect cardiac function. New technologies and methodologies will be required to identify sub-micron-sized structures, and engineering approaches must be undertaken for designing materials to enable the study of such small-scale properties on cellular behavior in vitro. As is often the case in life, small contributions can make a big difference.

## Data Availability

Not applicable.
